# A partitioned polygenic risk score reveals distinct contributions to psoriasis clinical phenotypes across a multi-ethnic cohort

**DOI:** 10.1186/s12967-024-05591-z

**Published:** 2024-09-11

**Authors:** Faye Orcales, Sugandh Kumar, Audrey Bui, Chandler Johnson, Jared Liu, Zhi-Ming Huang, Wilson Liao

**Affiliations:** 1 Department of Dermatology, University of California San Francisco, San Francisco, CA USA; 2 Lake Erie College of Osteopathic Medicine, Bradenton, FL USA; 3 Medical College of Georgia, Augusta University, Augusta, GA USA

## Abstract

Psoriasis is a chronic, immune-mediated inflammatory skin disease associated with a polygenic mode of inheritance. There are few studies that explore the association of a psoriasis Polygenic Risk Score (PRS) with patient clinical characteristics, and to our knowledge there are no studies examining psoriasis PRS associations across different ethnicities. In this study, we used a multi-racial psoriasis cohort to investigate PRS associations with clinical phenotypes including age of onset, psoriatic arthritis, other comorbidities, psoriasis body location, psoriasis subtype, environmental triggers, and response to therapies. We collected patient data and Affymetrix genome-wide SNP data from a cohort of 607 psoriasis patients and calculated an 88-loci PRS (PRS-ALL), also partitioned between genetic loci within the HLA region (PRS-HLA; 11 SNPS) and loci outside the HLA region (PRS-NoHLA; 77 SNPS). We used t-test and logistic regression to analyze the association of PRS with clinical phenotypes. We found that PRS-HLA and PRS-noHLA had differing effects on psoriasis age of onset, psoriatic arthritis, psoriasis located on the ears, genitals, nails, soles of feet, skin folds, and palms, skin injury as an environmental trigger, cardiovascular comorbidities, and response to phototherapy. In some cases these PRS associations were ethnicity specific. Overall, these results show that the genetic basis for clinical manifestations of psoriasis are driven by distinct HLA and non-HLA effects, and that these PRS associations can be dependent on ethnicity.

**Supplementary Information:**

The online version contains supplementary material available at 10.1186/s12967-024-05591-z.

## Introduction

Psoriasis is a chronic immune-mediated skin disease that affects roughly 3.6% of European ancestry groups, 2.5% of Asians, 1.9% of Hispanics, 1.5% of African Americans, and 3.1% of other/multiracial groups [1]. 90% of individuals with psoriasis experience it in the form of plaque psoriasis, also known as psoriasis vulgaris [2], and roughly 6–42% of psoriasis patients also experience psoriatic arthritis [3]. Psoriasis can negatively impact quality of life of those affected [4]. The prevalence of depression and anxiety in psoriasis patients is 20% and 21%, respectively [5]. Psoriasis is also known to be comorbid with various diseases including cardiovascular diseases, diabetes, metabolic syndrome, rheumatic conditions, and autoimmune diseases [4, 6–10].

The pathogenesis of psoriasis is characterized by dysregulation of the IL-17/23 axis and of various cell types including T-cells, dendritic cells, keratinocytes, and stromal cells [11]. The genetic contribution to psoriasis susceptibility has been estimated to be about 70% [12]. GWAS studies have found more than 70 susceptibility loci in the European population [13]. The HLA-C*06:02 allele is believed to contribute the largest genetic affect to psoriasis susceptibility. HLA-C*06:02 is associated with earlier onset of disease and increased likelihood of association with streptococcal throat infection [14–16]. While the discovery of psoriasis genetic variants has helped us further understand many genetic mechanisms in the pathogenesis of psoriasis, most genotype-phenotype studies have been conducted in European populations. Thus, we currently lack an understanding of the impact of psoriasis genetic variants on clinical features in non-European populations.

Polygenic Risk Scores (PRS) have increasingly been used to study various diseases with a polygenic mode of inheritance, like psoriasis. However, there are few studies that explore the association of psoriasis PRS with patient clinical characteristics, and there are no studies looking at PRS associations across different ethnicities. We calculated an 88-loci PRS on this multi-racial cohort, also subdivided between loci within the HLA region (HLA-PRS; 11 SNPS) and loci outside the HLA region (NonHLA-PRS; 77 SNPS). These 88 loci were derived from the largest psoriasis GWAS published to date [13]. We also calculated HLA-C*06:02 dosages for each patient to look at clinical associations with this specific allele. In our study, we sought to investigate PRS association with clinical phenotypes including age of onset, psoriatic arthritis, psoriasis body location, psoriasis subtype, environmental triggers for psoriasis, co-morbidities, and treatment response, all across a multi-ethnic cohort.

## Methodology

### Data collection

We collected patient-reported data, physician-reported data, and Affymetrix United Kingdom Biobank Axiom array SNP data from a cohort of 607 psoriasis patients enrolled at the University of California San Francisco (UCSF). The cohort consists of 381 patients with European ancestry, 148 Asian, and 78 Mixed/Non-European. Their mean age is 47 (SD = 17), with 46% being female and 54% male. Clinical data collected included maternal and paternal ethnicity, body mass index (BMI), age of onset, locations where psoriasis has ever presented, psoriasis type, triggers for psoriasis, co-morbidities, and response to treatments. Psoriatic arthritis (PsA) status was defined as follows: No PsA = No patient-reported joint pain and never diagnosed with PsA by a physician; Possible PsA = Patient-reported joint pain but never diagnosed with PsA by a physician; Confirmed PsA = Patient-reported joint pain and confirmed diagnosis of PsA by a rheumatologist. A complete list of clinical data fields collected can be found in Additional file 1: Supplementary Table S1. All patients were clinically diagnosed by a board-certified dermatologist and completed a written consent form for use of their data and biosamples under UCSF IRB#10–0230.

### Quality assessment and genotyping process

Before conducting polygenic risk calculations, we performed quality assessment of the genetic data in this study. All DNA samples were genotyped using the Affymetrix United Kingdom Biobank Axiom Array from ThermoFisher, employing the GeneTitan Multi-Channel Instrument by Applied Biosystems for genotyping. Subsequently, all genotyped variants underwent processing using Analysis Power Tools 2.10.2.2, which was provided by Affymetrix (source: https://www.affymetrix.com/support/developer/powertools/changelog/index.html). To ensure the high quality of the genotyped samples, we examined their DNA quality through a call rate exceeding 97% and Dish QC for genotyping quality threshold 82%. The resulting genotype variants were further analyzed using ‘snpflip’ (source: https://github.com/biocore-ntnu/snpflip) with the GRCh37 (also known as hg19) build of the human genome reference sequence (source: http://hgdownload.cse.ucsc.edu/goldenPath/hg19/bigZips/hg19.fa.gz) to address SNP flipping, reversal, and removal of ambiguous-stranded SNPs. Finally, the remaining sites were sorted using Plink 2.0 (source: www.cog-genomics.org/plink/2.0/) following the methodology outlined by Chang et al. in 2015 [17]. Next, all the genotyped data from individuals of different ethnicities was used for imputation on all 22 chromosomes for further polygenic risk analysis.

### Imputation of non-genotyped variants using the michigan imputation server

All genotyped data from samples representing three different ethnicities underwent imputation using the Michigan Imputation Server (source: https://imputationserver.sph.umich.edu) to impute non-genotyped variants with a reference dataset. Specifically, we employed the 1000G Phase 3 v5 reference panel based on GRCh37, with the rsqFilter deactivated, and utilized Eagle v2.4 for phasing within the European (EUR), Asian, and African populations. To ensure consistency and compatibility, the positions of the imputed SNPs were translated to GRCh38 coordinates using the ‘LiftoverVcf’ command available in Picard version 2.23.3 (source: http://broadinstitute.github.io/picard/). The quality of imputation was rigorously assessed and demonstrated a high level of accuracy with an R^2^ value exceeding 0.7, consistent with the recommendation from prior studies [18, 19]. Additional information about these SNPs is available in Additional file 1: Supplementary TableS2.

### SNP selection for polygenic risk score calculation

We used a total of 88 psoriasis susceptibility SNPs and their respective odds ratios (ORs) obtained from the largest published Genome-Wide Association Study (GWAS) meta-analysis of SNPs achieving genome-wide significance (*p* < 5.0E-08) [13]. The details of these selected SNPs can be found in Additional file 1: Supplementary TableS2. Further information on allele frequencies and imputation can be found in Additional file 2: Supplementary Tables S3-S4. This meta-analysis comprised data from six GWAS cohorts, one Exomechip study, and one Immunochip dataset, all focused on individuals of European ancestry. Importantly, many of the psoriasis loci identified in this study contained secondary independent signals. To accurately represent the effect sizes of the primary signals, adjustments were made by calculating the ORs while conditioning on the presence of other independent signals within the same genomic locus. This approach ensured that the independent contribution of each signal to the development of psoriasis was properly estimated, accounting for any linkage disequilibrium (LD) within the genomic structure. Furthermore, considering the notably high LD associated with the Human Leukocyte Antigen (HLA) loci, a pairwise linkage disequilibrium analysis was conducted to assess the LD between the 11 HLA SNPs across a European population. This analysis was performed using the LD-matrix Tool within LD-link, as previously described by Machiela and Chanock in 2015 [20]. The results of this analysis confirmed that there was low LD between the 11 selected HLA SNPs, with 93% of the pairwise LD comparisons exhibiting an R^2^ value of less than 0.1.

### Polygenic risk score calculations

As previously described, we computed the Polygenic Risk Score (PRS) by integrating the effects of all risk variants, considering their respective effect sizes, which represent the strength of the association between a genetic risk variant and the trait or condition of interest [21]. Further, our polygenic risk calculations were performed in three sets: (1) encompassing all 88 independent SNPs, (2) focusing on HLA SNPs (comprising 11 SNPs), and (3) focusing on non-HLA SNPs (comprising 77 SNPs). HLA SNPs were identified as those within the HLA region (located at chr6:28510120–33480577 in GRCh38). The separation of calculations into HLA and non-HLA subsets was motivated by the different genetic mechanisms involved, with HLA loci affecting antigen presentation and non-HLA loci generally affecting innate and adaptive immune activation. These polygenic risk calculations were carried out using Plink-2.0 tools using these options (Plink2 --vcf CombinedVcfFile ‘dosage = HDS’ --score SnpReference 4 6 7 ‘no-mean-imputation’ ‘head-read’ ‘list-variants’ --out CurrentOutputScoresPrefix).

### HLA-C*06:02 dosage

Moreover, we quantified the dosage of HLA-C*06:02 across the various ethnicities within our sample population. To achieve this, we conducted imputation for all genotyped variants located on chromosome 6 for each of the samples, through the MIS server with the same configuration employed for the PRS calculation.

### Association testing

We used t-test and generalized logistic/linear regression in R (v4.1.0) to analyze differences between clinical phenotypes. For the logistic regression analyses, three covariate terms were added to the equation (clinical phenotype ~ Age + Sex + Smoking Status + PRS). Metabolic syndrome was defined by a patient fulfilling at least 3 out of 5 criteria (BMI over 30, high blood pressure, high cholesterol, high triglycerides, and diabetes).

## Results

### Age of psoriasis onset

**Fig. 1:**
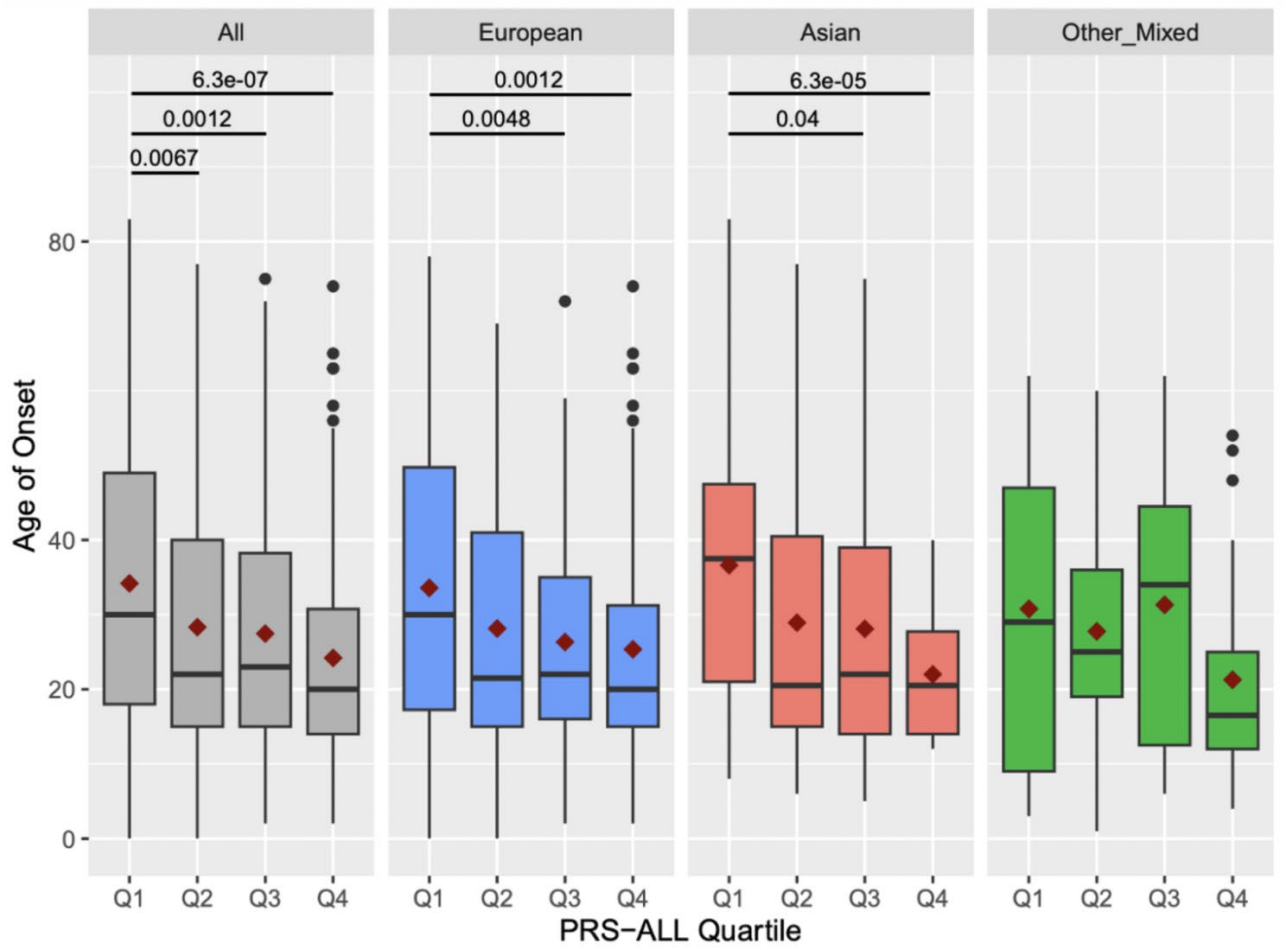
Age of psoriasis onset according to PRS-ALL quartiles. Each box plot is color-coded to show the age of onset distribution for each ethnicity: all ethnicities combined (gray), European (blue), Asian (red), other/mixed (green). The red diamond in a box plot represents the mean age of onset for that quartile group and the horizontal black line represents the median

Average age of onset was calculated for each PRS-ALL quartile across all ethnicity cohorts. The combined cohort, European cohort, and Asian cohort showed a consistent decreasing trend (Fig. 1). The combined cohort’s first quartile had an average age of onset of 34.19 (SD = 18.97), and its fourth quartile had an average age of onset of 24.20 (SD = 14.61). The European cohort’s first quartile had an average age of onset of 33.58 (SD = 19.17), and its fourth quartile had an average age of onset of 25.35 (SD = 15.52). The Asian cohort’s first quartile had an average age of onset of 36.62 (SD = 18.12), and its fourth quartile had an average age of onset of 22.00 (SD = 8.63). We also ran a mean-comparison t-test between each quartile group and each ethnic cohort. The combined ethnicity cohort showed that patients in PRS-ALL Q2, Q3, and Q4 all had significantly lower mean scores than patients in Q1. In the White and Asian cohorts, patients in PRS-ALL Q3 and Q4 had significantly lower mean scores than patients in Q1 (Fig. 1). These results indicate that increased total genetic burden as indicated by increasing PRS-ALL is associated with lower age of onset, with a magnified effect in the Asian cohort (Q4-Q1 difference ~ 15 years) compared to European cohort (Q4-Q1 difference ~ 8 years).

To understand the contribution of HLA versus non-HLA SNPs in driving this effect, the same analysis was done using PRS-HLA, HLA-C*06:02 status, and PRS-NoHLA. Results with PRS-HLA showed a consistent decreasing trend only in the combined and the European cohorts (Additional file 3: Figure S1). HLA-C*06:02-positive patients had significantly earlier average age of onsets for all ethnicity cohorts (Additional file 3: Figure S2). Results with PRS-NoHLA showed a decreasing trend with smaller magnitude starting at Q2 for the combined, European, and Other/Mixed cohort (Additional file 3: Figure S3). This indicates that HLA SNPs predominantly drive age of onset with the largest contribution from HLA-C*06:02, while non-HLA SNPs contribute a more moderate effect on age of onset.

### Psoriatic arthritis

**Fig. 2:**
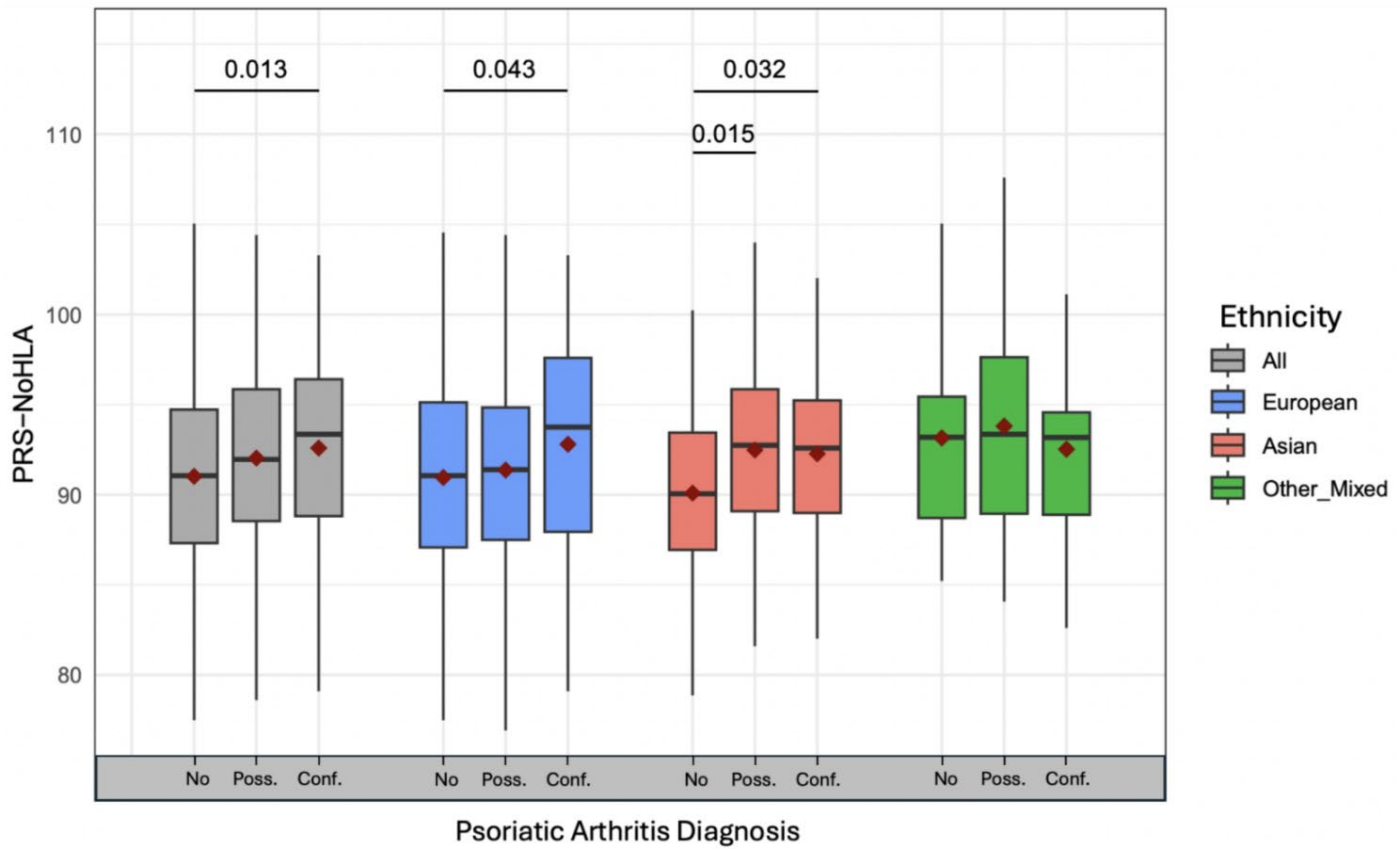
Psoriatic arthritis in relation to PRS-noHLA. Each box plot summarizes the PRS-NoHLA range for each ethnicity and PsA diagnosis group: all ethnicities combined (gray), European (blue), Asian (red), other/mixed (green). The x-axis labels correspond to each PsA diagnosis group: patients with no PsA (No), with possible PsA (Poss.), and with confirmed PsA (Conf.). The red diamond in a box plot represents the mean PRS-NoHLA for that diagnosis group and the horizontal black line represents the median. Mean comparison (t-test) p-values given at the top of plot, with lines indicating specific comparisons. Only significant p-values (< 0.05) are shown

To understand the contribution of PRS to psoriatic arthritis, a t-test was used to compare mean PRS between different psoriatic arthritis (PsA) phenotypes (no PsA, possible PsA, confirmed PsA). Comparisons tested with PRS-ALL and PRS-HLA scores were nonsignificant. However, PRS-NoHLA scores were found to be significantly higher in patients with confirmed PsA compared to patients with no PsA. This was seen in the combined cohort, and separately in the European and Asian cohorts (Fig. 2).

After excluding patients with possible PsA, logistic regression was performed to evaluate the effect of PRS on the outcomes “No PsA” and “Confirmed PsA”. Similar to the mean comparison results, PRS-ALL and PRS-HLA had nonsignificant results. PRS-NoHLA was significantly associated to PsA in the combined and European cohorts (Table 1). Together, the mean comparison and logistic regression results suggest that PRS-NoHLA is driving PsA, primarily in the European cohort.

Although PRS-HLA had nonsignificant results, logistic regression results with HLA-C*06:02 dosage revealed a significant protective association in the combined and European cohort. This indicates that HLA-C*06:02 may be protective for PsA amongst a cohort of individuals with psoriasis.

**Table 1:** Effects of PRS on psoriatic arthritis. Table contains odds ratios (ORs) and 95% confidence intervals calculated using a logistic regression model adjusted for age, sex, and smoking covariates. Cells with bolded values indicate significant p-values < 0.05, (+ if *p* < 0.05, ++ if *p* < 0.01)

Diagnosis	Ethnicity (*n* = No, Confirmed)	PRS-ALL OR (95% CI)	PRS-NoHLA OR (95% CI)	PRS-HLA OR (95% CI)	HLA-C*06:02 OR (95% CI)
PsA	European (200, 56)	1.04 (0.99–1.09)	**+ 1.07 (1.01–1.13)**	0.97 (0.88–1.06)	**+ 0.51 (0.27–0.94)**
	Asian (67, 35)	1.07 (0.98–1.18)	1.09 (0.99–1.19)	0.96 (0.85–1.10)	0.31 (0.09–1.07)
	Other/Mixed (30, 13)	0.99 (0.86–1.13)	0.97 (0.83–1.12)	1.05 (0.82–1.36)	1.19 (0.21–6.62)
	All (297, 104)	1.03 (0.99–1.07)	**+ 1.06 (1.01–1.10)**	0.96 (0.89–1.03)	**++ 0.45 (0.27–0.75)**

### Body locations ever affected by psoriasis

We evaluated the effect of PRS on body locations ever affected by psoriasis using logistic regression (Table 2). PRS-HLA was associated with psoriasis on the scalp in the European cohort but was protective against psoriasis on the genitals and on the nails in the Asian cohort. PRS-HLA was also protective against psoriasis appearing on the palms of hands for the combined, European, and Asian cohorts.

The HLA-C*06:02 allele had similar results to PRS-HLA for nail psoriasis, primarily in the Asian cohort. This indicates that this allele contributes the greatest effect in protection against psoriasis on the nails, for the Asian cohort.

PRS-NoHLA was significantly associated with psoriasis presence on the genitals, skin folds, ears, nails, and soles of feet. For all of these locations, PRS-NoHLA seemed to be driving psoriasis primarily in the European cohort. For nail psoriasis, PRS-NoHLA seemed to drive this mainly in the European and Asian cohorts.

Facial psoriasis was significantly associated with both PRS-ALL and PRS-NoHLA, only for the Other/Mixed cohort.

**Table 2:** Effects of PRS on body locations in which psoriasis has ever occurred. P-values were calculated using a logistic regression model adjusted for age, sex, and smoking covariates. Cells with bolded values indicate significant p-values < 0.05, (+ if *p* < 0.05, ++ if *p* < 0.01, +++ if *p* < 0.001)

Location	Ethnicity (*n* = unchecked, checked)	PRS-ALL OR (95% CI)	PRS-NoHLA OR (95% CI)	PRS-HLA OR (95% CI)	HLA-C*06:02 OR (95% CI)
Scalp	European (93, 271)	1.03 (0.99–1.06)	1.01 (0.97–1.05)	**+ 1.09 (1.01–1.18)**	1.08 (0.67–1.73)
	Asian (22, 123)	1.02 (0.93–1.12)	1.07 (0.97–1.17)	0.89 (0.77–1.03)	0.70 (0.24–2.05)
	Other/Mixed (10, 58)	1.07 (0.95–1.21)	1.08 (0.93–1.24)	1.04 (0.81–1.33)	2.65 (0.50-13.92)
	All (125, 452)	1.03 (0.99–1.06)	1.02 (0.99–1.06)	1.04 (0.97–1.10)	1.01 (0.67–1.51)
Ears	European (145, 219)	**+ 1.04 (1.01–1.07)**	**+ 1.04 (1.01–1.08)**	1.03 (0.96–1.10)	0.92 (0.61–1.40)
	Asian (63, 82)	1.05 (0.98–1.12)	1.06 (0.99–1.13)	0.97 (0.87–1.08)	1.77 (0.77–4.10)
	Other/Mixed (25, 43)	0.98 (0.89–1.06)	0.96 (0.87–1.06)	1.02 (0.86–1.23)	1.35 (0.46–3.96)
	All (233, 344)	**+ 1.03 (1.01–1.06)**	**+ 1.04 (1.01–1.07)**	1.02 (0.97–1.07)	1.14 (0.81–1.60)
Arms/ Legs	European (58, 306)	0.99 (0.95–1.03)	0.97 (0.92–1.01)	1.08 (0.99–1.18)	1.36 (0.76–2.42)
	Asian (18, 127)	1.00 (0.91–1.11)	1.07 (0.96–1.18)	0.86 (0.73-1.00)	0.56 (0.19–1.67)
	Other/Mixed (9, 59)	0.96 (0.84–1.10)	0.93 (0.80–1.07)	1.12 (0.83–1.49)	8.27 (0.65-105.85)
	All (85, 492)	0.99 (0.96–1.03)	0.98 (0.94–1.02)	1.02 (0.95–1.10)	1.21 (0.75–1.96)
Face	European (220, 144)	**+ 1.03 (1.00-1.06)**	1.03 (0.99–1.07)	1.06 (0.99–1.13)	1.18 (0.78–1.80)
	Asian (74, 71)	0.95 (0.89–1.02)	0.98 (0.91–1.04)	0.95 (0.84–1.06)	1.31 (0.57–3.02)
	Other/Mixed (41, 27)	**++ 1.15 (1.04–1.28)**	**++ 1.17 (1.04–1.31)**	1.07 (0.89–1.29)	0.99 (0.35–2.81)
	All (335, 242)	1.02 (0.99–1.05)	1.02 (0.99–1.05)	1.02 (0.97–1.07)	1.11 (0.79–1.56)
Genitals	European (246, 118)	**++ 1.05 (1.01–1.08)**	**+ 1.05 (1.01–1.09)**	1.07 (0.99–1.14)	0.97 (0.63–1.49)
	Asian (109, 36)	1.02 (0.94–1.10)	1.07 (0.99–1.16)	**+ 0.86 (0.75–0.99)**	0.36 (0.12–1.15)
	Other/Mixed (45, 23)	1.01 (0.93–1.11)	1.02 (0.93–1.13)	0.98 (0.81–1.18)	0.97 (0.34–2.80)
	All (400, 177)	**++ 1.04 (1.01–1.07)**	**++ 1.05 (1.02–1.08)**	1.03 (0.97–1.09)	0.94 (0.66–1.35)
Nails (pits/plaque)	European (234, 130)	**+ 1.03 (1.00-1.07)**	**+ 1.04 (1.00-1.08)**	1.02 (0.96–1.09)	0.76 (0.49–1.16)
	Asian (81, 64)	1.01 (0.94–1.08)	**+ 1.08 (1.01–1.16)**	**++ 0.83 (0.73–0.93)**	**++ 0.20 (0.07–0.56)**
	Other/Mixed (41, 27)	0.98 (0.90–1.07)	0.99 (0.90–1.10)	0.93 (0.77–1.13)	1.36 (0.46–4.06)
	All (356, 221)	1.02 (0.99–1.05)	**++ 1.04 (1.01–1.07)**	0.96 (0.91–1.01)	**++ 0.61 (0.43–0.87)**
Palms of hands	European (296, 68)	0.97 (0.94–1.01)	0.99 (0.95–1.04)	**++ 0.89 (0.81–0.97)**	+ 0.51 (0.29–0.90)
	Asian (109, 36)	1.00 (0.93–1.08)	1.06 (0.98–1.14)	**+ 0.85 (0.75–0.98)**	0.80 (0.31–2.08)
	Other/Mixed (58, 10)	1.02 (0.91–1.15)	1.03 (0.90–1.17)	1.01 (0.80–1.28)	1.47 (0.38–5.72)
	All (463, 114)	0.98 (0.95–1.01)	1.01 (0.97–1.04)	**+++ 0.88 (0.82–0.94)**	**+ 0.59 (0.37–0.92)**
Soles of feet	European (303, 61)	**+ 1.05 (1.00-1.09)**	**+ 1.06 (1.01–1.12)**	1.01 (0.92–1.10)	0.95 (0.54–1.65)
	Asian (114, 31)	0.99 (0.91–1.07)	1.02 (0.94–1.11)	0.90 (0.79–1.03)	0.45 (0.14–1.41)
	Other/Mixed (62, 6)	1.13 (0.97–1.31)	1.12 (0.94–1.33)	1.15 (0.86–1.53)	6.35 (0.99–40.53)
	All (479, 98)	1.03 (0.99–1.06)	+ 1.05 (1.01–1.09)	0.97 (0.91–1.04)	0.86 (0.55–1.35)
Armpit/ Groin/ Skin folds	European (231, 133)	**++ 1.04 (1.01–1.08)**	**++ 1.05 (1.01–1.09)**	1.03 (0.96–1.10)	1.08 (0.71–1.64)
	Asian (87, 58)	1.01 (0.94–1.08)	1.05 (0.98–1.12)	0.90 (0.80–1.01)	0.56 (0.24–1.33)
	Other/Mixed (45, 23)	1.04 (0.94–1.14)	1.07 (0.96–1.18)	0.93 (0.77–1.13)	0.75 (0.26–2.18)
	All (363, 214)	**++ 1.04 (1.01–1.06)**	**++ 1.05 (1.02–1.08)**	0.99 (0.94–1.04)	0.93 (0.66–1.31)

### Psoriasis subtype

Logistic regression analyses were also performed to evaluate the effect of PRS on psoriasis subtype (Table 3). PRS-HLA had a protective effect on pustular and erythrodermic psoriasis across the entire cohort.

The HLA-C*06:02 allele had similar results to PRS-HLA for both pustular and erythrodermic psoriasis. However, unlike PRS-HLA it did appear to drive guttate psoriasis across the entire cohort. This indicates that this allele contributes the greatest effect in protection against pustular and erythrodermic psoriasis, along with driving guttate psoriasis for the combined cohort.

Both PRS-ALL and PRS-NoHLA drove plaque psoriasis only in the Asian cohort.

**Table 3:** Effects of PRS on types of psoriasis. P-values were calculated using a logistic regression model adjusted for age, sex, and smoking covariates. Cells with bolded values indicate significant p-values < 0.05, (+ if *p* < 0.05, ++ if *p* < 0.01)

Type	Ethnicity (*n* = unchecked, checked)	PRS-ALL OR (95% CI)	PRS-NoHLA OR (95% CI)	PRS-HLA OR (95% CI)	HLA-C*06:02 OR (95% CI)
Plaque	European (67, 297)	0.98 (0.94–1.02)	0.98 (0.94–1.02)	0.98 (0.91–1.07)	0.75 (0.45–1.24)
	Asian (27, 118)	**+ 1.10 (1.00-1.20)**	**++ 1.13 (1.03–1.23)**	0.92 (0.80–1.06)	0.72 (0.27–1.93)
	Other/Mixed (17, 51)	0.97 (0.87–1.07)	1.02 (0.91–1.15)	0.81 (0.65–1.01)	0.47 (0.15–1.49)
	All (111, 466)	0.99 (0.96–1.02)	1.00 (0.97–1.04)	0.96 (0.90–1.02)	0.73 (0.49–1.10)
Guttate	European (261, 103)	1.03 (0.99–1.07)	1.02 (0.98–1.06)	1.07 (0.99–1.15)	1.45 (0.92–2.28)
	Asian (95, 50)	1.00 (0.93–1.07)	0.99 (0.92–1.06)	1.02 (0.91–1.14)	2.02 (0.89–4.62)
	Other/Mixed (48, 20)	0.97 (0.89–1.07)	0.93 (0.84–1.04)	1.12 (0.93–1.35)	2.71 (0.91–8.04)
	All (404, 173)	1.01 (0.99–1.04)	1.00 (0.97–1.03)	1.05 (0.99–1.11)	**+ 1.52 (1.07–2.16)**
Pustular	European (352, 12)	0.99 (0.91–1.08)	1.03 (0.93–1.14)	0.88 (0.73–1.07)	0.37 (0.10–1.44)
	Asian (134, 11)	0.96 (0.84–1.09)	1.04 (0.92–1.19)	0.80 (0.64–1.01)	0.49 (0.10–2.53)
	Other/Mixed (64, 4)	0.90 (0.74–1.11)	0.97 (0.78–1.20)	0.74 (0.46–1.18)	0.42 (0.04–4.29)
	All (550, 27)	0.97 (0.92–1.04)	1.03 (0.97–1.11)	**++ 0.80 (0.70–0.92)**	**+ 0.33 (0.13–0.86)**
Erythrodermic	European (359, 5)	0.97 (0.86–1.10)	0.98 (0.85–1.14)	0.89 (0.65–1.21)	0.00 (0.00-Inf)
	Asian (139, 6)	1.00 (0.84–1.19)	1.17 (0.97–1.41)	+ 0.60 (0.40–0.91)	0.00 (0.00-Inf)
	Other/Mixed (64, 4)	0.93 (0.77–1.13)	0.98 (0.81–1.20)	0.76 (0.48–1.20)	1.03 (0.09–12.09)
	All (562, 15)	0.98 (0.90–1.06)	1.05 (0.96–1.15)	**++ 0.75 (0.61–0.91)**	**+ 0.12 (0.02–0.96)**
Inverse (Armpit/ Groin)	European (331, 33)	0.97 (0.91–1.03)	0.99 (0.92–1.06)	0.89 (0.78–1.03)	0.55 (0.21–1.45)
	Asian (137, 8)	0.94 (0.72–1.22)	1.01 (0.76–1.34)	0.85 (0.56–1.31)	1.42 (0.10-19.39)
	Other/Mixed (63, 5)	1.00 (0.00-Inf)	1.00 (0.00-Inf)	1.00 (0.00-Inf)	1.00 (0.00-Inf)
	All (531, 46)	0.97 (0.91–1.03)	0.99 (0.92–1.06)	0.90 (0.79–1.03)	0.70 (0.29–1.71)

### Environmental triggers for psoriasis

Logistic regression analyses were also performed to evaluate the effect of PRS on environmental triggers for psoriasis (Table 4). Both PRS-ALL and PRS-HLA had a significant effect on the strep throat and winter season triggers. PRS-HLA drove strep throat as a psoriasis trigger in just the combined and Other/Mixed cohorts. For the winter season trigger, PRS-HLA showed a driving effect for all cohorts except the Asian cohort. PRS-HLA had a significant protective association with the medication trigger, only in the Asian cohort.

The HLA-C*06:02 allele had similar results to PRS-HLA for the strep throat trigger, only in the combined cohort. However, unlike PRS-HLA, HLA-C*06:02 had a significant association with strep throat in the European cohort. This indicates that this allele contributes the greatest effect in driving strep throat as a trigger primarily within the European cohort. HLA-C*06:02 also showed a significant association with stress (physical, emotional, psychological) that was not shown in PRS-HLA. It seemed to be driving stress, primarily in the other/mixed cohort. This may indicate that primarily HLA-C*06:02 SNPs are driving stress as a psoriasis trigger in non-European and non-Asian individuals.

PRS-NoHLA was significantly associated with skin injury (Koebner phenomenon), and summer season (sunlight). It was associated with these psoriasis triggers, mostly in the European cohort.

PRS-ALL, PRS-NoHLA, and PRS-HLA all seemed to drive Winter season as a trigger for psoriasis, primarily within the European cohort.

**Table 4:** Effects of PRS on environmental triggers for psoriasis. P-values were calculated using a logistic regression model adjusted for age, sex, and smoking covariates. Cells with bolded values indicate significant p-values < 0.05, (+ if *p* < 0.05, ++ if *p* < 0.01, +++ if *p* < 0.001)

Environmental Trigger	Ethnicity (*n* = unchecked, checked)	PRS-ALL OR (95% CI)	PRS-NoHLA OR (95% CI)	PRS-HLA OR (95% CI)	HLA-C*06:02 OR (95% CI)
Stress (Physical, Emotional, Psychological)	European (118, 246)	1.03 (0.99–1.06)	1.03 (0.99–1.07)	1.04 (0.97–1.11)	1.11 (0.71–1.72)
	Asian (35, 110)	1.05 (0.96–1.13)	1.04 (0.96–1.13)	1.02 (0.89–1.16)	1.42 (0.47–4.31)
	Other/Mixed (17, 51)	1.02 (0.93–1.13)	1.00 (0.90–1.12)	1.10 (0.89–1.36)	**+ 6.16 (1.12–33.89)**
	All (170, 407)	**+ 1.03 (1.00-1.06)**	1.03 (0.99–1.06)	1.03 (0.97–1.09)	1.19 (0.81–1.73)
Skin Injury	European (260, 104)	**+++ 1.07 (1.03–1.10)**	**+++ 1.09 (1.05–1.14)**	1.00 (0.93–1.07)	0.83 (0.52–1.30)
	Asian (115, 30)	1.02 (0.93–1.10)	1.00 (0.92–1.09)	1.04 (0.91–1.18)	1.05 (0.40–2.76)
	Other/Mixed (51, 17)	0.97 (0.88–1.07)	0.95 (0.85–1.07)	1.01 (0.84–1.23)	1.47 (0.48–4.48)
	All (426, 151)	**++ 1.05 (1.02–1.08)**	**++ 1.06 (1.02–1.09)**	1.02 (0.96–1.08)	1.01 (0.70–1.47)
Strep Throat	European (323, 41)	1.03 (0.98–1.08)	1.02 (0.96–1.08)	1.08 (0.97–1.20)	**++ 2.43 (1.26–4.69)**
	Asian (134, 11)	1.03 (0.90–1.17)	0.98 (0.87–1.11)	1.11 (0.92–1.35)	3.08 (0.84–11.37)
	Other/Mixed (60, 8)	1.16 (0.99–1.35)	1.01 (0.88–1.17)	**++ 1.73 (1.20–2.51)**	**+ 5.99 (1.25–28.63)**
	All (517, 60)	**+ 1.05 (1.00-1.10)**	1.01 (0.96–1.06)	**++ 1.15 (1.05–1.25)**	**+++ 2.93 (1.74–4.95)**
Winter Season	European (152, 212)	**++ 1.05 (1.02–1.08)**	**+ 1.04 (1.01–1.08)**	**++ 1.10 (1.03–1.18)**	1.36 (0.89–2.07)
	Asian (67, 78)	0.99 (0.92–1.06)	0.97 (0.90–1.04)	1.05 (0.93–1.18)	1.19 (0.50–2.83)
	Other/Mixed (36, 32)	0.99 (0.91–1.08)	0.93 (0.84–1.03)	**+ 1.22 (1.01–1.48)**	2.85 (0.92–8.80)
	All (255, 322)	**++ 1.03 (1.01–1.06)**	1.02 (0.99–1.05)	**+++ 1.10 (1.04–1.16)**	**+ 1.45 (1.03–2.05)**
Summer Season (Sunlight)	European (340, 24)	**+ 1.08 (1.02–1.15)**	**++ 1.11 (1.03–1.19)**	1.02 (0.90–1.16)	0.86 (0.38–1.96)
	Asian (132, 13)	0.93 (0.82–1.06)	0.97 (0.85–1.10)	0.90 (0.73–1.10)	0.32 (0.04–2.63)
	Other/Mixed (63, 5)	1.03 (0.87–1.22)	1.06 (0.88–1.29)	0.93 (0.65–1.35)	0.00 (0.00-Inf)
	All (535, 42)	1.04 (0.99–1.09)	1.06 (0.99–1.12)	0.99 (0.90–1.10)	0.68 (0.34–1.35)
Medications	European (340, 24)	0.98 (0.93–1.04)	0.97 (0.91–1.04)	1.01 (0.89–1.15)	1.39 (0.63–3.06)
	Asian (140, 5)	0.96 (0.81–1.15)	1.10 (0.91–1.31)	**+ 0.63 (0.42–0.96)**	0.00 (0.00-Inf)
	Other/Mixed (64, 4)	1.06 (0.89–1.26)	1.05 (0.86–1.28)	1.10 (0.79–1.53)	**+ 17.06 (1.25-232.19)**
	All (544, 33)	0.99 (0.94–1.05)	1.00 (0.94–1.06)	0.99 (0.89–1.10)	1.64 (0.84–3.20)

### Psoriasis co-morbidities

Logistic regression analyses were also performed to evaluate the effect of PRS on co-morbidities (Table 5). PRS-HLA had significant negative associations with high blood pressure and eczema. For high blood pressure this was primarily seen in the European cohort, and for eczema this was seen mostly in the Asian cohort.

Like PRS-HLA, HLA-C*06:02 was found to be protective against high blood pressure mainly in the European cohort. This indicates that the HLA-C*06:02 allele contributed the greatest effect in protecting against high blood pressure.

PRS-ALL and PRS-NoHLA seemed to drive metabolic syndrome for the combined and Other/Mixed cohorts.

**Table 5:** Effects of PRS on co-morbidities present in psoriasis patients. P-values were calculated using a logistic regression model adjusted for age, sex, and smoking covariates. Cells with bolded values indicate significant p-values (+ if *p* < 0.05)

Co-morbidity	Ethnicity (*n* = unchecked, checked)	PRS-ALL OR (95% CI)	PRS-NoHLA OR (95% CI)	PRS-HLA OR (95% CI)	HLA-C*06:02 OR (95% CI)
High Blood Pressure	European (275, 89)	1.00 (0.97–1.03)	1.01 (0.98–1.05)	**+ 0.93 (0.88–0.99)**	**+ 0.57 (0.33–0.99)**
	Asian (96, 49)	0.98 (0.95–1.02)	1.00 (0.95–1.04)	0.93 (0.86–1.01)	1.05 (0.42–2.65)
	Other/Mixed (49, 19)	1.04 (0.96–1.12)	1.04 (0.97–1.12)	1.00 (0.88–1.13)	1.10 (0.25–4.97)
	All (420, 157)	1.09 (0.96–1.23)	1.15 (0.98–1.34)	0.96 (0.74–1.23)	**+ 0.61 (0.40–0.95)**
High Cholesterol	European (274, 90)	0.99 (0.96–1.02)	0.99 (0.96–1.03)	0.97 (0.91–1.03)	1.11 (0.66–1.86)
	Asian (108, 37)	0.98 (0.95–1.02)	0.97 (0.93–1.01)	1.01 (0.93–1.09)	0.44 (0.13–1.51)
	Other/Mixed (56, 12)	1.04 (0.96–1.13)	1.06 (0.97–1.15)	0.96 (0.84–1.11)	0.46 (0.08–2.81)
	All (438, 139)	1.04 (0.91–1.18)	1.14 (0.96–1.34)	0.77 (0.55–1.07)	0.86 (0.55–1.32)
Stroke	European (358, 6)	0.96 (0.88–1.04)	0.98 (0.89–1.08)	0.87 (0.71–1.05)	0.42 (0.05–3.82)
	Asian (142, 3)	0.93 (0.83–1.04)	0.91 (0.80–1.03)	0.97 (0.75–1.27)	0.00 (0.00-Inf)
	Other/Mixed (64, 4)	0.89 (0.68–1.18)	1.34 (0.87–2.07)	0.00 (0.00-Inf)	0.00 (0.00-Inf)
	All (564, 13)	1.04 (0.86–1.26)	1.04 (0.84–1.29)	1.04 (0.70–1.55)	0.21 (0.03–1.68)
Eczema	European (357, 7)	0.94 (0.86–1.02)	0.96 (0.87–1.07)	0.83 (0.67–1.02)	0.00 (0.00-Inf)
	Asian (142, 3)	0.92 (0.83–1.02)	0.95 (0.84–1.07)	**+ 0.71 (0.53–0.97)**	2.03 (0.17–24.41)
	Other/Mixed (67, 1)	1.08 (0.85–1.37)	1.06 (0.83–1.34)	1.05 (0.72–1.51)	0.00 (0.00-Inf)
	All (566, 11)	0.89 (0.60–1.32)	0.75 (0.39–1.45)	1.10 (0.55–2.23)	0.19 (0.02–1.50)
Metabolic Syndrome	European (335, 29)	1.00 (0.96–1.04)	1.02 (0.97–1.07)	0.94 (0.86–1.03)	0.89 (0.39–2.02)
	Asian (131, 14)	0.96 (0.91–1.01)	0.95 (0.89–1.02)	0.96 (0.85–1.09)	1.25 (0.31-5.00)
	Other/Mixed (59, 9)	**+ 1.15 (1.01–1.31)**	**+ 1.17 (1.02–1.34)**	0.99 (0.82–1.19)	0.35 (0.04–3.54)
	All (525, 52)	**+ 1.18 (1.00-1.39)**	**+ 1.30 (1.04–1.61)**	0.97 (0.72–1.30)	0.77 (0.40–1.46)

### Treatment response

Logistic regression analyses were also performed to evaluate the effect of PRS on treatment response outcomes “Responder” and “Non-Responder” (Table 6). The “Responder” outcome was assigned to patients who self-reported either a slightly better or much better response to a treatment. The “Non-Responder” outcome was assigned to patients who self-reported no effect or a worse response to a treatment.

PRS-HLA was associated with a positive response to Narrowband UVB treatment and Combined UVB treatments in the combined cohort. PRS-NoHLA was negatively associated with a positive response to PUVA treatment, primarily in the Asian cohort. The HLA-C*06:02 allele did not show any significant associations for either of these treatments.

**Table 6:** Effects of PRS on psoriasis treatment response. P-values were calculated using a logistic regression model adjusted for age, sex, and smoking covariates. For certain analyses marked with an *, smoking was taken out as a covariate due to no presence of smokers in the data for that specific treatment and ethnic group. Cells with bolded values indicate significant p-values < 0.05, (+ if *p* < 0.05, ++ if *p* < 0.01)

Treatment	Ethnicity (*n* = Non, Resp)	PRS-ALL OR (95% CI)	PRS-NoHLA OR (95% CI)	PRS-HLA OR (95% CI)	HLA-C*06:02 OR (95% CI)
Narrowband UVB	European (10, 114)	1.01 (0.91–1.11)	0.96 (0.86–1.07)	1.30 (0.99–1.69)	1.93 (0.51–7.24)
	Asian (9, 61)	0.99 (0.85–1.15)	0.90 (0.76–1.06)	1.25 (0.96–1.62)	2.22 (0.24–20.80)
	*Other/Mixed (1, 21)	5.40 (0.00-Inf)	1.48 (0.33–6.63)	2.75 (0.00-Inf)	4.52 (0.00-Inf)
	All (20, 196)	1.01 (0.94–1.09)	0.95 (0.88–1.04)	**++1.26 (1.06–1.50)**	2.22 (0.77–6.39)
UVB Treatments (Broadband, Narrowband, Other)	European (22, 175)	1.05 (0.98–1.12)	1.04 (0.97–1.11)	1.12 (0.96–1.31)	1.51 (0.62–3.70)
	Asian (17, 91)	0.95 (0.85–1.06)	0.88 (0.78–0.99)	1.21 (1.00-1.46)	2.14 (0.43–10.59)
	Other/Mixed (5, 33)	1.08 (0.90–1.29)	1.03 (0.84–1.27)	1.22 (0.84–1.77)	3.40 (0.30-38.06)
	All (44, 299)	1.03 (0.98–1.08)	0.99 (0.94–1.05)	**+ 1.16 (1.04–1.30)**	1.90 (0.94–3.85)
PUVA	European (10, 49)	1.01 (0.91–1.10)	0.99 (0.89–1.10)	1.05 (0.85–1.30)	2.40 (0.55–10.58)
	*Asian (6, 23)	**+0.73 (0.55–0.96)**	0.39 (0.15–1.01)	1.09 (0.78–1.53)	4.01 (0.00-Inf)
	*Other/Mixed (3, 7)	1.06 (0.89–1.26)	0.99 (0.79–1.24)	1.70 (0.72–4.02)	4.75 (0.00-Inf)
	All (19, 79)	0.97 (0.91–1.04)	0.93 (0.85–1.01)	1.12 (0.96–1.32)	4.41 (1.12–17.32)

**Fig. 3:**
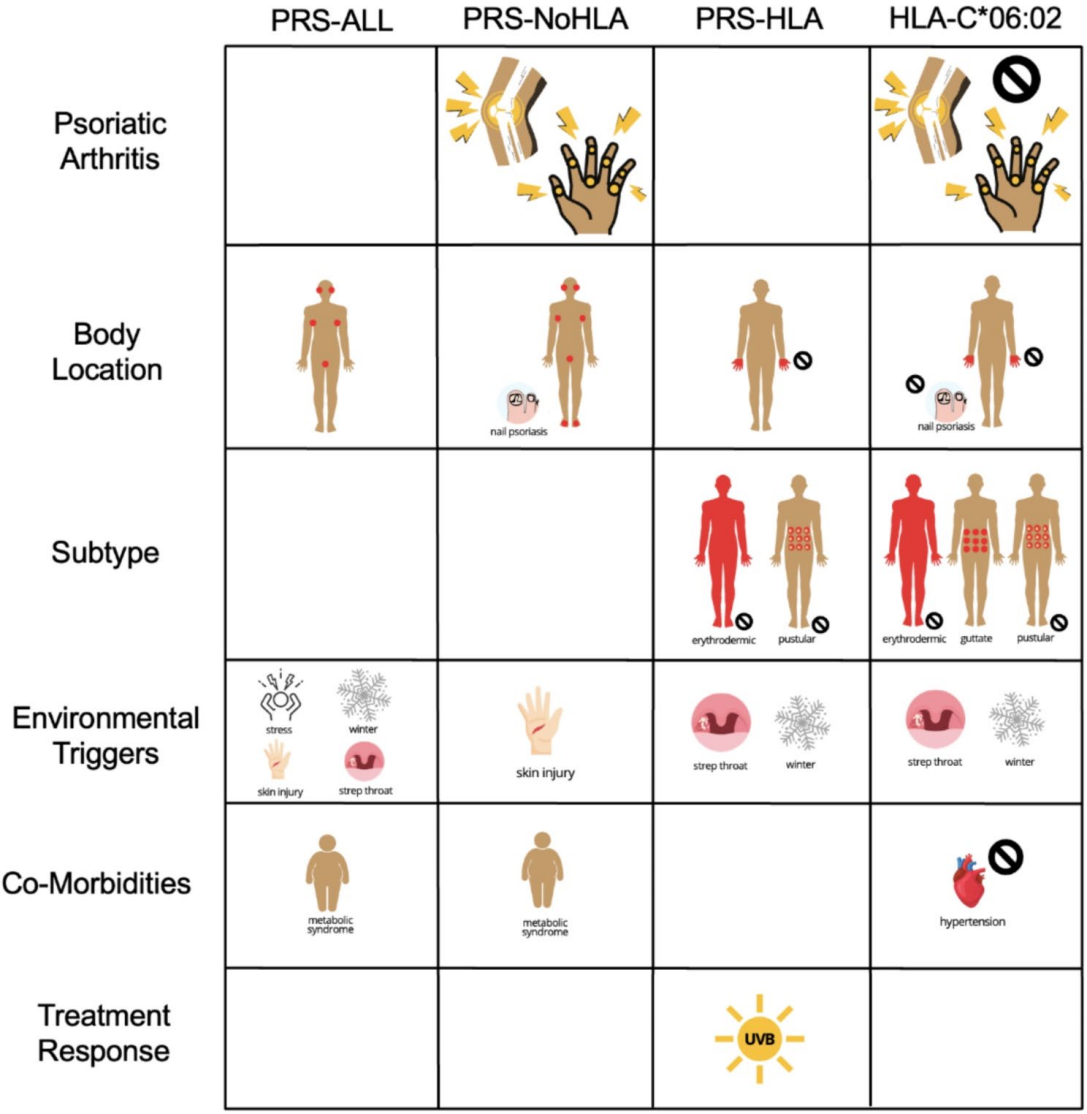
Logistic regression summary of significant PRS associations across several clinical phenotypes in the combined cohort. For body location, red spots indicate an association at the respective location, with the exception of nail psoriasis which is shown zoomed in and on the bottom left. Psoriasis subtypes were represented with individually labelled body figures. PRS associations with psoriatic arthritis, environmental triggers, co-morbidities, and treatment response were represented with simple images. Red spots or images with a black cross represent a protective effect (OR < 1) for that specific phenotype, and images without a black cross represent a driving effect

## Discussion

There are few studies that explore the association of psoriasis PRS with comprehensive patient characteristics, and there are no studies looking at PRS across different ethnicities. In this study we evaluated PRS-ALL, PRS-noHLA, PRS-HLA, and HLA-C*06:02 on age of onset, psoriatic arthritis, psoriasis body location, psoriasis subtype, psoriasis environmental triggers, co-morbidities, and treatment response in different racial groups.

For age of onset, we found that the European and Asian cohorts had a consistent decreasing trend across PRS-ALL quartiles. Furthermore, HLA SNPs may be driving this trend primarily for the European cohort. This agrees with previous published findings [21–25]. We did not see this trend for HLA SNPs in our Asian cohort. However, a study which calculated a 14-loci PRS on a Han Chinese cohort did find that their group with a high-risk PRS-HLA had a significantly earlier age of onset than their low-risk group [26]. While we did not find PRS-HLA to associate with age of onset in our Asian cohort, we did find that HLA-C*06:02 was associated with lower age of onset in our Asian cohort (Additional file 3: Figure S2). Moreover, the HLA-PRS we calculated for our Asian cohort used a set of psoriasis susceptibility loci identified from a European population which could contribute to this discrepancy.

Our PsA results indicate that non-HLA SNPs are driving PsA, primarily in the European cohort. Previous studies have identified up to 50 non-HLA susceptibility genes of PsA in various European cohorts, many of which are inflammation-related genes that activate or inhibit signaling from *IL23A*,* TNIP1*,* ERAP1*,* ERAP2*,* IL12B* [27]. A mean comparison t-test did reveal that Asian patients with confirmed PsA had significantly higher PRS-NoHLA scores than Asian patients without PsA. One study in a Chinese cohort found six non-HLA psoriasis susceptibility loci that were significantly associated with PsA [28]. The candidate genes for these loci were *TNIP1*,* IL28RA*,* IL12B*,* ERAP1*,* PTTG1*, and *GJB2*. Another study that used a different Chinese PsA cohort confirmed three non-HLA susceptibility loci. The candidate genes for these loci were *IL12B*,* RUNX3*, and *LCE* [29]. We also found that HLA-C*06:02 specific SNPs may be protective against PsA in our combined cohort. This is consistent with findings from a previous study done on a European cohort, which found that PsA was observed less frequently in HLA-C*06:02-positive patients compared to HLA-C*06:02-negative patients [30]. A previous study done on a Han Chinese population found no significant difference between HLA-C*06:02-negative and -positive patients regarding PsA [31].

Our findings on psoriasis body location for the European cohort indicated that HLA SNPs were primarily driving psoriasis on the scalp. The association between HLA SNPs and scalp psoriasis is still unclear. Findings for our Asian cohort showed that HLA SNPs may be protective against psoriasis on the genitals, nails, and palms. A study done in a Han Chinese population showed that palmoplantar pustulosis was observed in HLA-C*06:02-negative patients [31]. Our result showing that HLA SNPs may be protective against genital psoriasis in an Asian cohort is a novel finding. Our findings also showed that non-HLA SNPS were driving psoriasis on the genitals, skin folds, nails, ears, and soles of feet, primarily in the European cohort. Non-HLA SNPS also seemed to drive psoriasis on the face, primarily in the Other/Mixed cohort. These findings are novel, as currently there are a lack of studies investigating the role of non-HLA genetic factors on these body locations.

Our psoriasis subtype results showed that HLA-C*06:02 was driving guttate psoriasis in the combined cohort. This is consistent with previous studies which found that guttate psoriasis was more frequent in HLA-C*06:02-positive patients [14, 23, 30]. Results also indicated that HLA SNPs were protective for pustular and erythrodermic psoriasis in the combined cohort. Prior studies have found that HLA-C*01:02-positivity has been associated with a higher frequency of erythrodermic psoriasis in a Chinese cohort and higher frequency of pustular psoriasis in a Japanese cohort [32, 33]. We did not specifically examine HLA-C*01:02 in our cohorts. We also found that non-HLA SNPs may be driving plaque psoriasis, primarily in our Asian cohort.

The interaction between environmental triggers and genetic susceptibility is not well understood. Our data showed that for almost all significant associations between PRS and environmental triggers, they had an OR > 1.0. This indicates that HLA and non-HLA SNPs may be driving certain environmental factors as triggers for psoriasis. Various studies have reported HLA-C*06:02 to be associated with strep throat, which is consistent with our findings [14–16]. Two studies did not find a significant difference between HLA-C*06:02-positive and negative patients regarding psoriasis exacerbation during stress, which stands in contrast with our findings [14, 31]. One of those studies also found that HLA-C*06:02-positive patients had a higher incidence of Koebner’s phenomenon, which describes the appearance of new skin lesions in the same area where a skin injury occurred [14]. This conflicts with our results in which we found no significant association between HLA-C*06:02 and skin injury as a trigger. Instead, we found a significant association with non-HLA SNPs with skin injury, primarily in European patients. This could be due to a difference in cohorts. Gudjonsson et al.’s cohort was comprised of only Icelandic patients; therefore, their cohort is more genetically isolated than our cohort of patients from UCSF. Their cohort also had a higher proportion of HLA-C*06:02-positive patients (654/1019 = 0.64) than our cohort (221/607 = 0.36). Furthermore, their HLA-C*06:02-positive patients with a late age of onset were associated with mild psoriasis, while their HLA-C*06:02-negative patients with a late age of onset were associated with more severe psoriasis. We observed the opposite pattern in our combined and European HLA-C*06:02-negative cohorts (Additional file 3: Figure S4). Our result showing HLA SNPs driving winter season as an environmental trigger in non-European patients is a novel finding.

Many studies on psoriasis cardiometabolic comorbidities have focused mainly on its relationship with the HLA-C*06:02 allele. HLA-C*06:02-positive status is often associated with lower central adiposity, lower incidence of hypertension, and a lower prevalence of other cardiometabolic comorbidities [30, 34, 35]. This was consistent with one of our HLA-C*06:02 results, which was found to be protective against high blood pressure. Our results also showed that HLA SNPs were protective against eczema primarily in our Asian cohort. Our results also showed that non-HLA SNPs are associated with metabolic syndrome in Other/Mixed ethnicities.

Our results for treatment response indicate that PRS-ALL is associated with “No Response” to PUVA treatment in our Asian cohort. However, this finding did result from a small sample size (*n* < 25). Our results also showed that HLA SNPs are associated with positive response to Narrowband UVB (NB-UVB). NB-UVB had the same direction of effect for HLA-C*06:02 (but non-significant). In contrast, another study found that HLA-C*06:02-positive patients experienced reduced effectiveness with NB-UVB therapy [36]. This conflicting result could be due to a difference in cohorts. Bojko et al. analyzed a cohort of 306 Polish Caucasian psoriasis patients. Their cohort also had a higher proportion of HLA-C*06:02-positive patients (181/306 = 0.59) than our cohort (221/607 = 0.36).

There were a number of limitations within our study including using a limited number of disease associated variants, limited transferability of PRS in difference ethnicities, and limited clinical variables. Since we only used a certain number of variants in our PRS calculations, our scores are not able to explain total disease heritability. Furthermore, there is a dearth of psoriasis GWAS studies done on non-European populations other than East Asians. Therefore, it is not fully known whether common disease associated variants identified in European populations are transferable to non-European populations, although one trans-ethnic meta-analysis suggested considerable overlap [37]. Future methods that can be used to deal with this limitation include re-calculation of PRS on full summary statistics and using more robust methods such as PRS-CSx and PTRS [38, 39]. Our patient data also does not include information on change in PASI, diet, and allergies. These are topic of interests that can hopefully be covered in future studies.

In conclusion, the results of this study showed that PRS have significant associations with a large number of psoriasis clinical phenotypes (Fig. 3), and these associations can show different results for different ethnicities. We would like to highlight the importance of several identified non-HLA associations, which differs from the focus on HLA associations in the literature. Non-HLA SNPs were associated with PsA in our European and Asian cohorts. Also, non-HLA SNPs drove the presence of psoriasis on the genitals, skin folds, nails, ears, and soles of feet, primarily in European patients. Non-HLA SNPs was associated with skin injury (Koebner phenomenon) as a trigger for psoriasis, primarily in European patients. Finally, non-HLA SNPs were associated with metabolic syndrome, primarily in non-European, non-Asian ethnicities. This study advances our knowledge of psoriasis endotypes, wherein genetic factors influence how psoriatic disease presents, is triggered, and responds to therapy.

## Electronic supplementary material

Below is the link to the electronic supplementary material.

Supplementary Material 1

Supplementary Material 2

Supplementary Material 3

## Data Availability

The datasets analyzed during the current study are not publicly available due to genetic privacy protections but are available from the corresponding author upon reasonable request.
