# Supplementary material for: A partitioned polygenic risk score reveals distinct contributions to psoriasis clinical phenotypes across a multi-ethnic cohort
Source: J Transl Med. 2024 Sep 11;22:835. doi: 10.1186/s12967-024-05591-z (PMC11389070; doi:10.1186/s12967-024-05591-z)
Supplement: Supplementary file 3 — Supplementary Material 3 [file 12967_2024_5591_MOESM3_ESM.docx]

Supplementary Information – Additional File 3

**
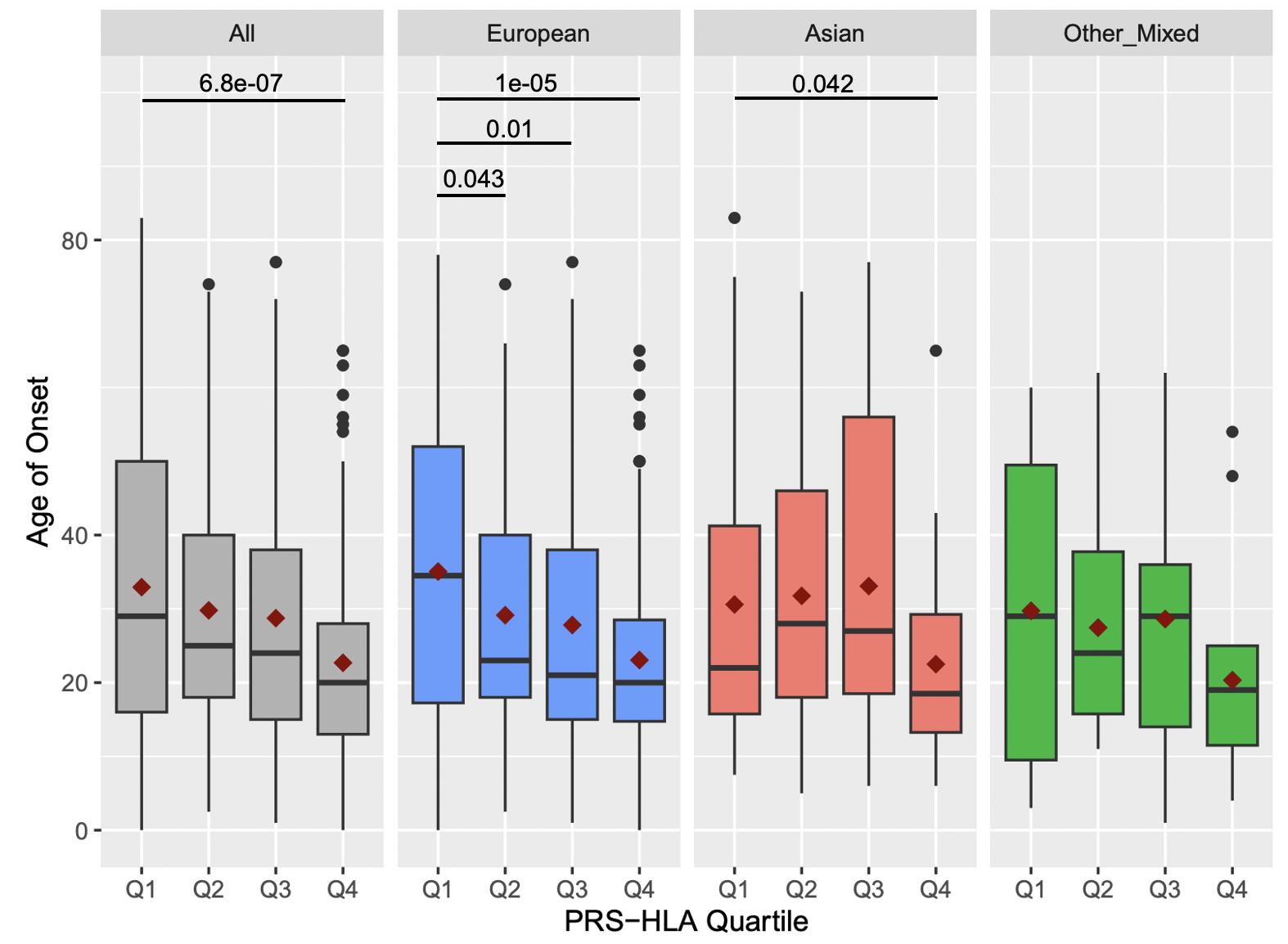
**

**Figure S1. Age of psoriasis onset according to PRS-HLA quartiles.** Each box plot is color-coded to show the age of onset distribution for each ethnicity: all ethnicities combined (gray), European (blue), Asian (red), other/mixed (green). The red diamond in a box plot represents the mean age of onset for that quartile group and the horizontal black line represents the median.

**
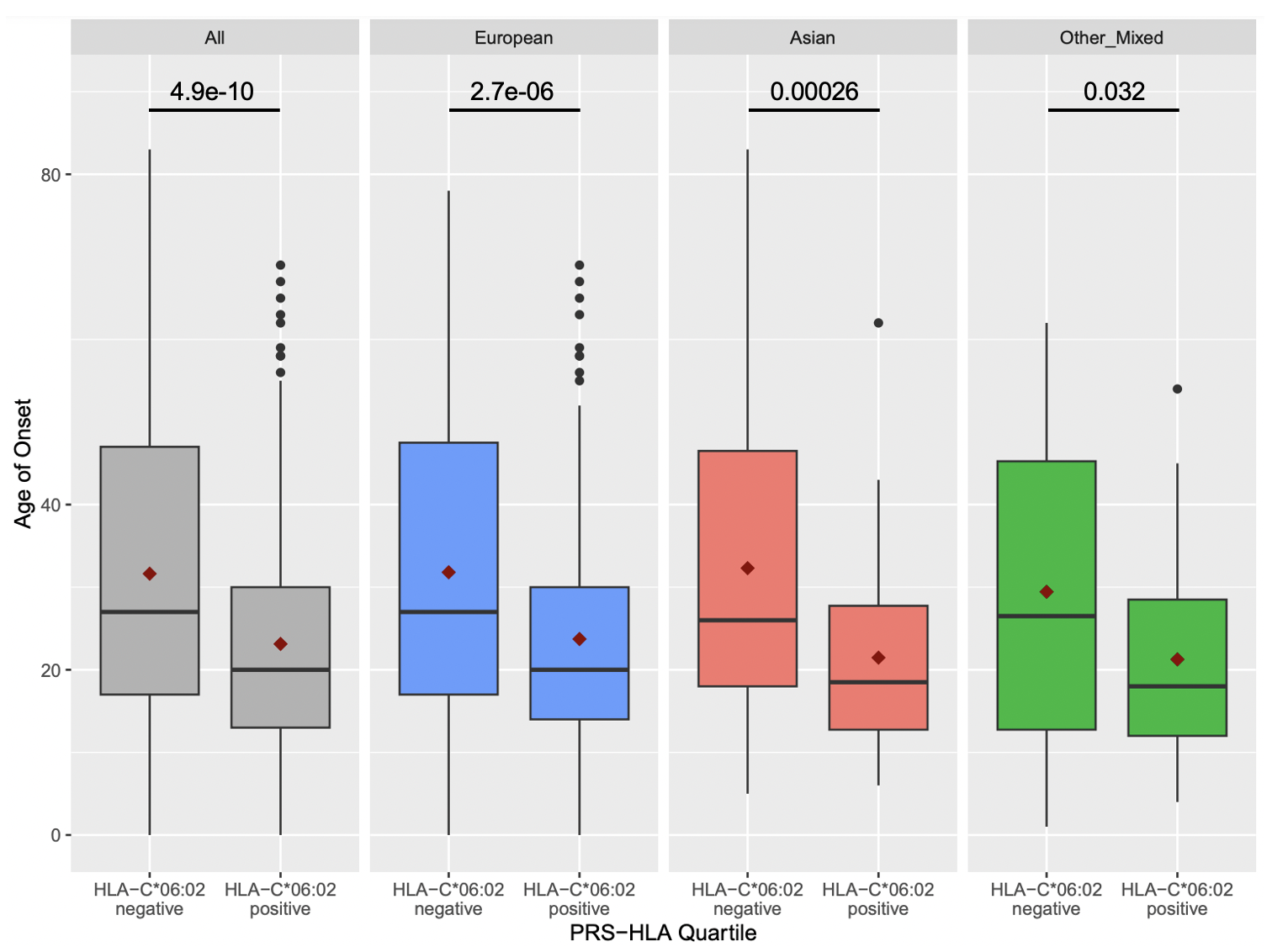
**

**Figure S2. Age of psoriasis onset according to HLA-C*06:02 status.** Each box plot is color-coded to show the age of onset distribution for each ethnicity: all ethnicities combined (gray), European (blue), Asian (red), other/mixed (green). The red diamond in a box plot represents the mean age of onset for that quartile group and the horizontal black line represents the median.


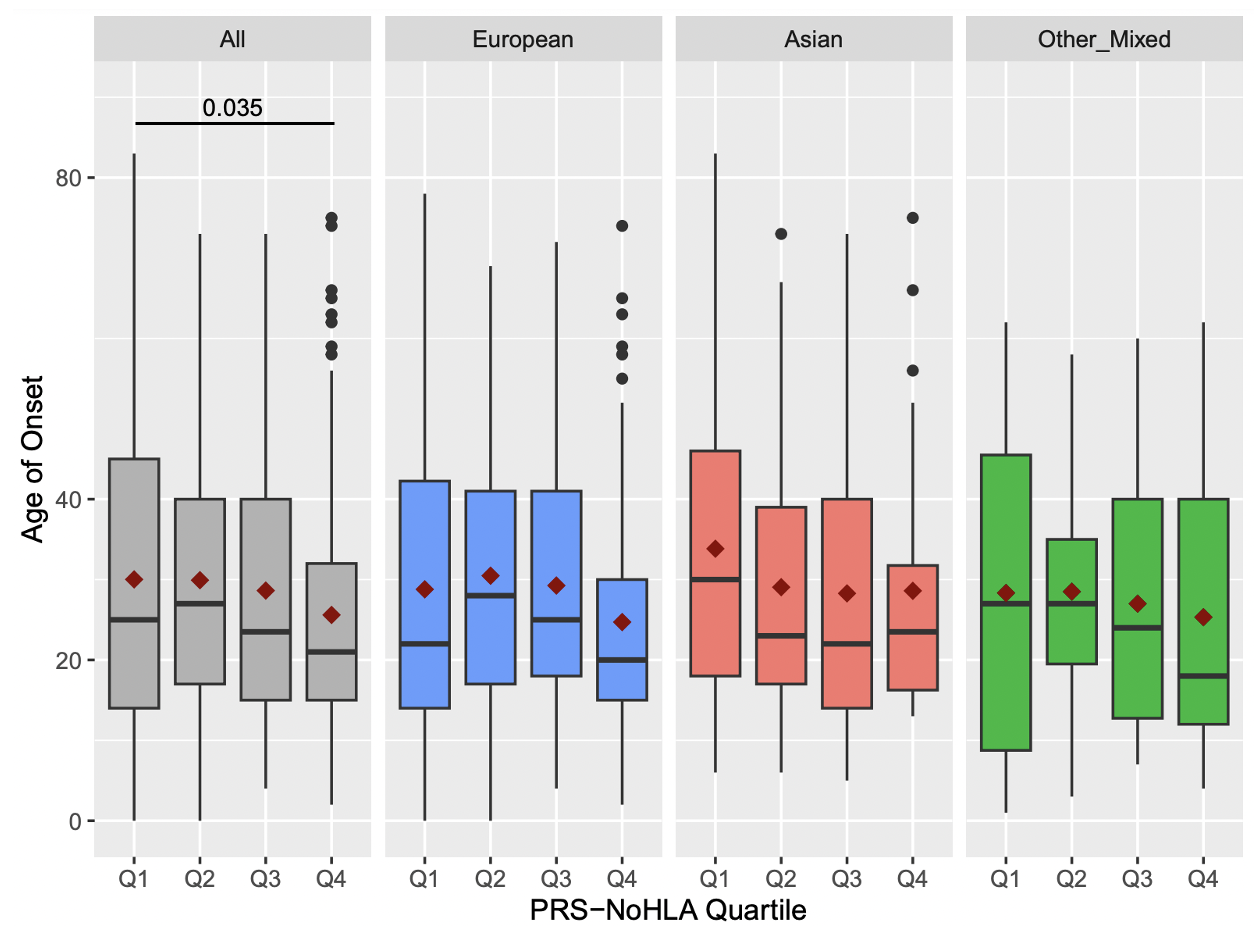


**Figure S3. Age of psoriasis onset according to PRS-NoHLA quartiles.** Each box plot is color-coded to show the age of onset distribution for each ethnicity: all ethnicities combined (gray), European (blue), Asian (red), other/mixed (green). The red diamond in a box plot represents the mean age of onset for that quartile group and the horizontal black line represents the median.


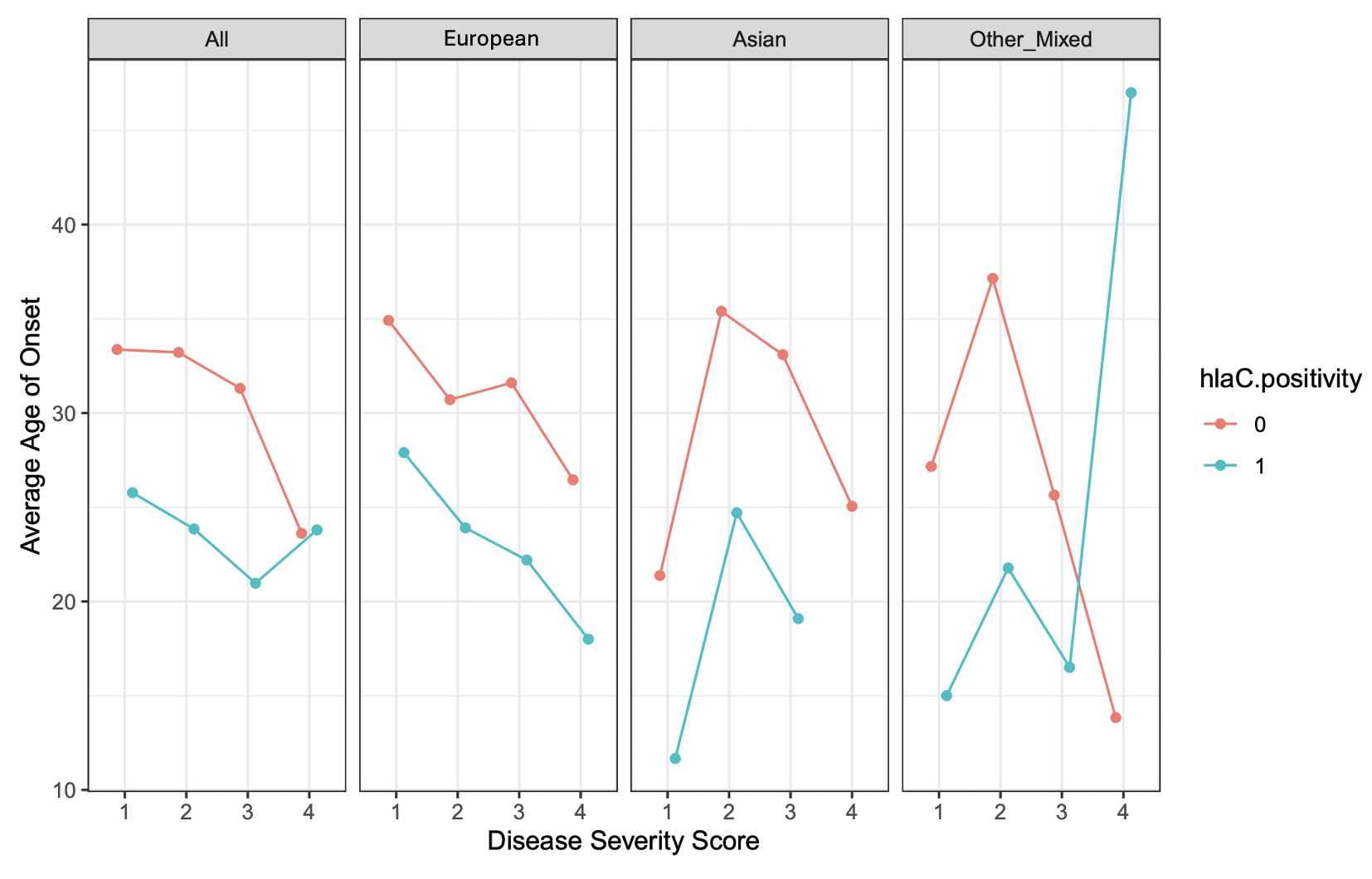


**Figure S4. Average age of psoriasis onset according to disease severity.** Each point represents the mean age of onset according to severity levels for each race. Severity levels were scored according to the following: 1 (mild), 2 (moderate), 3 (severe), 4 (very severe). Mean age of onset values were calculated for each *HLA-C*06:02* status: *HLA-C*06:02*-negative (pink),

*HLA-C*06:02*- positive (blue).
